# Timely Review and Communication of Histopathology Reports Following Appendicectomy: Insights from a Two-Cycle Clinical Audit

**DOI:** 10.7759/cureus.58539

**Published:** 2024-04-18

**Authors:** Christine-Bianca Hanganu, Sanad Isswiasi, Abiodun Adigun, Vladimir Nichita, Rishi Sen, Muhammadhasan Anwaar, Elisabeth Drye

**Affiliations:** 1 General Surgery, Addenbrooke's Hospital, Cambridge University Hospitals NHS Foundation Trust, Cambridge, GBR; 2 General Surgery, West Suffolk Hospital, West Suffolk NHS Foundation Trust, Bury St Edmunds, GBR; 3 Cardiothoracic Surgery, Wythenshawe Hospital, Manchester University NHS Foundation Trust, Wythenshawe, GBR; 4 General Surgery, Peterborough City Hospital, North West Anglia NHS Foundation Trust, Peterborough, GBR

**Keywords:** documented patient communication, patient safety guidelines, nhs guidelines, histopathology, appendicectomy, appendicitis, acute surgical unit, histology post appendicectomy

## Abstract

Introduction: Appendicectomy is the most frequent emergency general surgical procedure. Prior research highlights the importance of histopathology analysis after appendicectomy which is the practice in many countries including the United Kingdom (UK), aiming to prevent any oversight of vital findings and the avoidance of potential delays in patient care. Our primary objective was to audit the extent to which surgeons adhere to the NHS England patient safety guidelines from 2016 when it comes to timely reviewing and effectively communicating histopathology results to patients and/or their general practitioners following appendicectomy procedures. Our secondary objective was to amend practice, if deemed necessary, following the implementation of agreed-upon protocols, with the expected improvements being observable in the second cycle of the audit.

Methods: In our two-cycle audit, we performed a retrospective analysis using online patient records from a single centre in the UK. The initial cycle involved cases of emergency appendectomies carried out consecutively for suspected appendicitis from April 2018 to June 2019. Following the clinical governance meeting and the implementation of recommendations, the second audit cycle covered cases between September 2020 and October 2020.

Results: In the first cycle, among 418 laparoscopic appendectomies, 207 (49.52%) and 47 reports (11.24%) were reviewed within a 15-day and a 16-30-day window, respectively, following the online availability of histopathology results. Notably, 116 reports (27.75%) remained unreviewed by surgeons, and only 67 (16.02%) of these reports documented communication with patients and/or their general practitioners. In the second cycle, involving 49 patients, 38 reports (77.55%) were reviewed within the first 15 days, and 10 reports (20.4%) were reviewed between 16-30 days. Among these, 16 reports (32.65%) documented communication with patients and/or their general practitioners.

Conclusions: Our adherence to the aforementioned guidance was poor prior to this audit. This two-cycle audit highlighted the need for improvement in the timely review and communication of histopathology reports following appendectomy at our centre. The second cycle showed promising progress, suggesting that changes implemented between the cycles had a positive impact. Nevertheless, continuous efforts may be required to enhance and sustain adherence to these vital patient safety guidelines.

## Introduction

Appendicectomy is the most frequent emergency general surgical procedure. Prior research highlights the importance of routine histopathology analysis after appendicectomy [1,2]. This widely adopted practice, observed in many countries including the United Kingdom (UK), is essential for gaining invaluable insights into the pathological characteristics of the appendix. It not only facilitates accurate diagnosis but also helps to identify other potentially significant pathologies [1-3].

In addition, managing unexpected findings in appendicectomy specimens involves a broad spectrum of options, ranging from no further intervention to more invasive measures including staging, bowel resection, adjuvant therapy, and surveillance. This underscores the crucial importance of timely knowledge of histology in certain situations [4-6]. Moreover, in a healthcare setting, overlooking a crucial result can have substantial implications for patient safety and may lead to serious medico-legal issues for healthcare providers [7,8].

In 2016, the NHS England Patient Safety Domain [9], supported by the Academy of Medical Royal Colleges, recommended that hospital clinical teams implement a robust consultant-led protocol to ensure the prompt and efficient management of test results. This includes ensuring that test results are reviewed, necessary actions are taken, and effective communication and follow-up are facilitated. Our primary objectives were to examine the extent to which surgeons adhere to the NHS England patient safety guidelines when it comes to reviewing and effectively communicating histopathology results following appendicectomy in our centre. Our secondary objective was to modify practices, if required, after implementing agreed-upon protocols, with the anticipated improvements being noticeable in the second audit cycle.

## Materials and methods

A two-cycle retrospective clinical audit was conducted at Peterborough City Hospital, Peterborough, UK. We included consecutive cases of emergency laparoscopic appendectomies performed for suspected appendicitis during the periods of April 2018 to June 2019 and September 2020 to October 2020, respectively. Patient characteristics, including age and gender, were recorded. We documented the timing of histopathology reports becoming available online and when surgeons reviewed them post appendectomy using our local Sunquest ICE database (Sunquest Information Systems Inc., Tucson, Arizona, United States). This system enabled us to determine who reviewed the reports and when, with clear timestamps for each review.

Additionally, we examined communication with patients and general practitioners via eTrack (Teegearment SG PTE. LTD, Singapore), another online platform for correspondence. Our criteria were established based on the NHS England Patient Safety Domain's standards from 2016. These recommendations advocate for the discharging team, led by a consultant, to ensure that test results are promptly reviewed, acted upon, and effectively communicated to both general practitioners and patients. This should also include follow-up and future plans. Data were processed in an Excel 365 spreadsheet (Microsoft Corporation, Redmond, Washington, United States). Findings from both cycles were discussed in clinical governance meetings, leading to implemented changes between cycles. This project was authorised by the Quality Governance & Compliance Department at Peterborough City Hospital (approval number: 3208).

## Results

In the first cycle, we examined 418 appendicectomies and this included 210 males and 208 females (1:1) with a median age of 25. The median postoperative stay was one day. We found that 207 cases (49.52%) had their histopathology reports reviewed within 15 days following the online availability of the histology reports. An additional 47 reports (11.24%) were reviewed within 16-30 days, 46 reports (11%) within 31-90 days, and two reports (0.48%) took more than 90 days to be reviewed. Among the histopathology reports reviewed, 279 (92.38%) were assessed solely by the discharging consultants. However, there were 116 reports (27.75%) that had never been reviewed by a surgeon prior to the audit. Regarding documented communication of histopathology results to patients and/or their general practitioners, only 67 reports (16.02%) were communicated to patients and/or general practitioners via letters in the first audit cycle.

In the second cycle, following the implementation of an agreed-upon protocol within the department, we examined 49 appendectomies. This included 25 males and 24 females (1:1) with a median age of 29. The median postoperative hospital stay was two days. Of these 49 appendicectomies, 38 histopathology reports (77.55%) were reviewed within 15 days, while an additional 10 reports (20.4%) were reviewed between 15-30 days post the online availability of the histology. Notably, 32.65% of these reports documented communication with patients and/or general practitioners.

**Figure 1:**
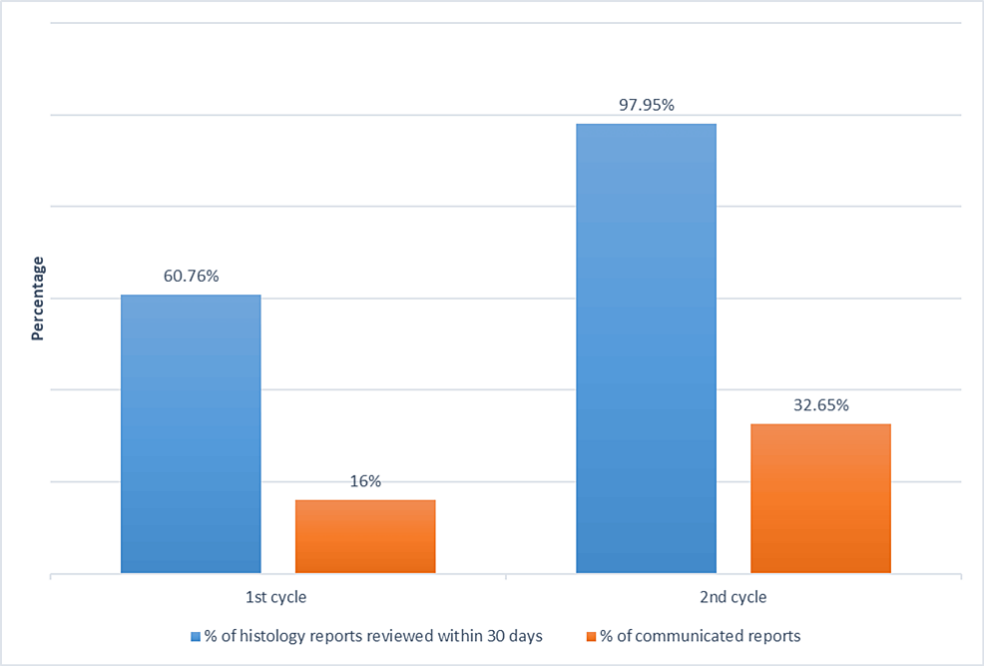
Main outcomes from the first and the second audit cycles. Communicated reports indicate reports that documented communication with patients and/or general practitioners

Table 1 summarises the patient characteristics of both cycles. The median time observed to publish the online report was equally seven days in the two cycles. Table 2 and Figure 1 summarise our findings.

**Table 1:** Summary of patient characteristics. Data represented as N and median (where mentioned)

	1^st^ cycle	2^nd^ cycle
Period	April 2018 to June 2019	September 2020 to Octoer 2020
Number of patients	418	49
Male:Female	210:208	25:24
Median age (years)	25 years	29 years
Median postoperative stay in days	1	2
Median number of days to publish report	7	7

**Table 2:** Summary of the findings. Data represented as N (%) Communicated reports indicate reports that documented communication with patients and/or general practitioners

	1^st^ cycle, n (%)	2^nd^ cycle, n (%)
Normal appendix in histopathology report	106 (25.35%)	9 (18.36%)
Histology reviewed in 0-15 days	207 (49.52%)	38 (77.55%)
Histology reviewed in 16-30 days	47 (11.24%)	10 (20.40%)
Histology reviewed in 31-90 days	46 (11%)	1 (2%)
Histology reports reviewed within 30 days	254 (60.76%)	48 (97.95%)
Grade of the doctor who reviewed the report:		
Consultant only	279 (92.38%)	25 (51%)
Registrar only	13 (4.3%)	1 (2%)
Both	10 (3.31%)	23 (46.9%)
Communicated reports	67 (16.02%)	16 (32.65%)

## Discussion

In the UK, it is standard practice to send appendiceal specimens for histopathological analysis. This practice is considered essential as relying solely on laparoscopy has the potential to overlook significant pathologies [1-3,10]. This oversight is primarily attributed to the reduced tactile sensitivity of laparoscopy when compared to direct hand palpation, further compounded by the challenges posed by inflammation, which can obscure the detection of lesions. Studies have also reported that the incidence of malignant appendiceal tumours has significantly risen [11-13]. The reported increase in malignant appendiceal tumours, especially those involving neuroendocrine tumours (NETs), may reflect improved histopathological analysis rather than a surge in actual incidence [12].

Failure to acknowledge significant test results can result in serious health implications and may lead to allegations of medical negligence [9,14-16]. A UK-based study by Mosedale and colleagues reported that 2% of the medicolegal claims after appendicectomy were related to inadequate follow-up resulting in a median payout of £8,205 per case by the NHS between 2002 and 2011 [16]. They also found a lack of appropriate response to histological reports indicating malignancy, resulting in a 100% success rate in litigation. They suggested that implementing local protocols is essential to ensure prompt action and follow-up for all specimens with abnormal histopathology. Evidence suggests that communication or system-related failures, rather than clinical negligence, often serve as primary triggers for claims [17,18].

According to healthcare experts, to establish efficient and coordinated follow-up for abnormal results, it is crucial to designate staff responsibilities, implement standardised processes for result identification and management, and employ information technology to ensure a closed-loop system [19-21]. The first step towards improvement should involve clarifying staff roles for each test type. In the context of our audit, the three key players are the ordering provider, the pathology department responsible for generating results, and the clinical team (surgeons) overseeing and communicating abnormalities. Ambiguities in responsibility often lead to care gaps.

The results from the first cycle of the audit indicate that the timely review of histopathology reports following appendicectomy, along with adherence to the NHS England Patient Safety Domain's standards, is suboptimal. Only 49.52% of histopathology reports were reviewed within 15 days, with 60.76% reviewed within a month. An important observation was that 27.75% of histopathology reports had not been acknowledged by surgeons before the first cycle as we were the first to review the reports during our audit, and it's reassuring that no significant pathology was missed. Furthermore, our findings revealed that only 16.02% of the reports were evidently communicated with patients and/or their general practitioners following their operation. This implies that most patients may experience delays in receiving essential information about their diagnosis, potentially affecting their treatment and long-term care.

As discussed during our departmental audit meeting, these findings led to the suggestion of involving registrars in the process of receiving and responding to results. Consultants exclusively assessed the majority (92.38%) of the reviewed histopathology reports in the first cycle, highlighting the limited participation of registrars in the review process. Registrars, who conduct independent appendectomies, were advised to select "send ICEmail," ensuring that the results are received by two colleagues, namely the responsible consultant and the registrar who requested the histology. Recommendations also proposed reviewing histopathology reports within four weeks and implementing periodic email reminders and posters for the team as some registrars stay for a few months only during their rotation. Additionally, it was suggested that patients should not be removed from the consultants’ inpatient active list until the histology report has been reviewed and communicated by a team member. Apart from improving patient safety, the department believes that involving registrars in such clinical protocols would benefit them educationally and professionally, exposing them to a wider range of conditions and increasing their engagement with patients they have operated on.

Furthermore, to monitor progress and identify areas for improvement, we recommended conducting a re-audit. This follow-up audit enabled us to track the effectiveness of the implemented changes and make further adjustments if needed. In fact, the second cycle of the audit showed promising progress, with 77.55% of histopathology reports being reviewed within 15 days, with about 98% of reports being reviewed within a month. It is notable that nearly half of the reports (47%) were acknowledged jointly by a consultant and a registrar. This collaborative acknowledgement signifies a shared responsibility between experienced consultants and trainee registrars. This improvement indicates that the interventions implemented between the cycles have positively influenced the timely review process and heightened awareness among surgeons.

Nevertheless, the 32.65% of documented communication in the second cycle, while an improvement, falls short of the desired level. It's worth noting that a considerable number of surgeons reported traditionally informing patients about laparoscopy findings in person after the operation on the ward round. Some expressed reservations about the necessity of writing a letter, particularly when the histopathology report indicates a normal appendix.

The limitations of our audit include its retrospective nature and its restriction to a single centre. Furthermore, the second cycle's sample size is smaller compared to the first, and it was conducted during the coronavirus disease 2019 (COVID-19) pandemic, which may not reflect the typical NHS working environment. However, we believe that the topic is of paramount importance, and our findings merit attention with the potential for broader examination and application. To our knowledge, the post-appendectomy review of histology reports by surgeons, particularly within the NHS context, has remained unexplored. Our audit may carry implications for improving follow-up and ensuring patient safety and satisfaction after appendicectomy.

## Conclusions

Our adherence to the aforementioned guidance was poor prior to this audit. This two-cycle audit highlighted the need for improvement in the timely review and communication of histopathology reports following appendectomy at our centre. The second cycle showed promising progress, suggesting that changes implemented between the cycles had a positive impact. Nevertheless, continuous efforts may be required to enhance and sustain adherence to these vital patient safety guidelines.
